# Depth-aware pose estimation using deep learning for exoskeleton gait analysis

**DOI:** 10.1038/s41598-023-50207-z

**Published:** 2023-12-19

**Authors:** Yachun Wang, Zhongcai Pei, Chen Wang, Zhiyong Tang

**Affiliations:** School of Automation Science and Electrical Engineering, Beihang University, Beijing, 100191 China

**Keywords:** Computer science, Software

## Abstract

In rehabilitation medicine, real-time analysis of the gait for human wearing lower-limb exoskeleton rehabilitation robot during walking can effectively prevent patients from experiencing excessive and asymmetric gait during rehabilitation training, thereby avoiding falls or even secondary injuries. To address the above situation, we propose a gait detection method based on computer vision for the real-time monitoring of gait during human–machine integrated walking. Specifically, we design a neural network model called GaitPoseNet, which is used for posture recognition in human–machine integrated walking. Using RGB images as input and depth features as output, regression of joint coordinates through depth estimation of implicit supervised networks. In addition, joint guidance strategy (JGS) is designed in the network framework. The degree of correlation between the various joints of the human body is used as a detection target to effectively overcome prediction difficulties due to partial joint occlusion during walking. Finally, a post processing algorithm is designed to describe patients’ walking motion by combining the pixel coordinates of each joint point and leg length. Our advantage is that we provide a non-contact measurement method with strong universality, and use depth estimation and JGS to improve measurement accuracy. Conducting experiments on the Walking Pose with Exoskeleton (WPE) Dataset shows that our method can reach 95.77% PCKs@0.1, 93.14% PCKs@0.08 and 3.55 ms runtime. Therefore our method achieves advanced performance considering both speed and accuracy.

## Introduction

In recent years, the aging trend of the world population is contributing to an increasing number of patients with lower limb motor dysfunction. With progress in science and technology and improvements in worldwide medical rehabilitation, determining how to safely and efficiently conduct rehabilitation training of lower limb motor function has become a research hotspot in the field of rehabilitation medicine^1,2^.Rehabilitation lower-limb exoskeleton robots can provide assistance and protection for daily rehabilitation training of patients. Gait evaluation in human–machine collaborative walking is an important aspect of rehabilitation training evaluation. If patients who walk with exoskeletons are impacted by excessive, asymmetric and unstable gaits, they may suffer from falls and even secondary damage due to low energy consumption^3^. Therefore, to improve the safety of human–machine cooperation, the gait must be analyzed accurately when the human–machine system is cooperating to walk. At the same time, it is helpful for doctors to intuitively understand the walking status of patients and adjust the process arrangement of rehabilitation training in a timely manner.

Gait evaluation methods include time parameters, kinematic and dynamic parameters, and EMG activity data, among others. The existing methods is mainly based on wearable sensors detection, which is a contact based measurement method. By placing sensors on subjects’ feet, knees, or hips, gait features are obtained. The main sensor technologies include inertial measurement units (IMUs)^4–7^, pressure sensors^8–10^, and electromyography (EMG)^11–13^. However, as contact-based measurement methods, these can cause discomfort to patients during testing and negatively impact their normal gait. These wearable sensors need to be connected to embedded devices to send signals and read data. These technologies require professional technicians to analyze gait data recorded on embedded devices, which gives the methods poor versatility.

Visual technology based on deep learning is now widely used in various fields. For example, in the field of industrial production, visual technology can be used to achieve faster and more accurate detection and identification of products, reducing human and material resources, and improving production efficiency and quality. In the medical field, by detecting medical images such as CT (computed tomography) scans and MRI (magnetic resonance imaging), suspected lesion areas can be quickly and accurately located and labeled, helping doctors make diagnosis and treatment decisions. In the field of autonomous driving, various sensing methods such as cameras, millimeter wave radar and LiDAR (lightlaser detection and ranging) are used to obtain information about target objects such as roads, vehicles and pedestrians, which can achieve high-precision object detection and tracking. Based on this, we propose to apply human pose estimation technology to the field of exoskeleton gait evaluation, providing a non-contact measurement method.

DeepPose^14^ first introduced convolutional neural network(CNN) in the field of human posture estimation in 2014, and the traditional template matching method is replaced with the CNN to regress the coordinates of the joint. This major breakthrough has landmark significance for the development of human pose estimation (HPE). The successive emergence of Stacked Hourglass^15^, HRNet^16^, OpenPose^17^, and AlphaPose^18^ has further established the position of CNN in HPE. The model mentioned above is categorized as 2D HPE. It lacks dimensional information and spatial generalization ability, which can cause the loss of spatial information on the feature maps. 3D HPE^19–22^, is more accurately describes human pose recognition and behavior and has higher research significance than 2D HPE. The depth images can effectively filter out the effects of clothing color, wrinkles, and other factors on RGB images, and the depth images are not affected when by poor lighting of the shooting scene. However, 3D HPE will design models with larger parameter quantities to achieve relative spatial coordinate output. To achieve non-contact measurement of exoskeleton gait, we propose a neural network model called GaitPoseNet for posture detection. To achieve accurate and fast measurement, we propose GaitPoseNet using only RGB image as network input and depth feature as network output (only predicting relative depth).

In summary, the highlights of this research are as follows: (1) we propose a multimodality human pose estimation model, GaitPoseNet, which predicts joint points and depth feature from RGB image. Assist in supervised regression of joint points through deep information. (2) Joint guidance strategy (JGS) is proposed based on the characteristics of human–machine system collaborative walking, which takes the degree of correlation between each joint as one of the detection targets. Through directional guidance, it efficiently captures the features of occluded joint points and solves the problem of partial joint occlusion caused by single camera photography. (3) A post processing algorithm is designed to analyze the gait by using the pixel coordinates of each joint point detected by GaitPoseNet and the leg length of subjects. The walking motion is described by geometric features. The geometric features of gait include joint rotation angle, stride length, spatiotemporal parameters of gait and gait symmetry. (4) We provide an RGB-D dataset with fine annotation on the detection of human walking joint points, including 7176 RGB images and corresponding depth images of dozens of experimenters walking freely and wearing the exoskeleton in different scenes.

The remaining sections of this article are arranged as follows: “Related works” introduces some common HPE models and some neural network models that take depth feature for prediction. “Methods” describes the proposed method, including the following: (1) GaitPoseNet, a neural network model with RGB images as input and depth feature as output. (2) JGS proposed based on the characteristics of human–machine system collaborative walking. (3) The loss function based on multitask loss. (4) The post processing algorithm for converting joint points into geometric features of gait. “Experiments” presents experimental results that validate the gait analysis performance of the proposed method in human–machine system collaborative walking scene. “Discussion” expands on the discussion. “Conclusion concludes the paper.

## Related works

In this section, we mainly focus on two aspects, namely the related works on human pose estimation as non-contact measurement and depth estimation.

### Pose estimation methods

As the foundation of human action recognition, HPE has great potential in analyzing human motion and capturing tiny pose information that the human eye easily overlooks. With the popularity of deep learning, HPE has become a research hotspot attracting much attention from scholars.

Single human pose estimation is the foundation of HPE, which mostly uses supervised regression methods. Single HPE can be divided into coordinate regression and heatmap regression. In reference^14,23^, CNN is used to directly regress the joint point coordinates. CNN has relatively fast detection speed, but it lacks spatial generalization ability, loses spatial information on the feature map, and has a fully connected layer that is easy to overfit. Therefore, other detection methods based on heatmap regression are used. In reference^24–26^, the heatmap detection method is used to obtain the probability distribution and location information of the joint points. This method usually has higher prediction accuracy than coordinate regression. For multiperson pose estimation, top-down model is proposed in reference^18,27^that first detects the human bounding box and then performs single pose estimation within the bounding box. Because each detection is divided into two steps, the real-time performance of the detection is poor, and the boundary box positioning error will directly lead to a decline in the accuracy of the method. In reference^14,15,17^, bottom-up model is proposed. This method has faster processing speed than the top-down method, but its accuracy is greatly affected by complex backgrounds and human occlusion. In reference^28^, an efficient method for gait analysis based on video detection is proposed that uses 2D video as input to extract skeletal joints.

3D HPE adds depth information and describes the poses more accurately than 2D HPE. In addition, it has a wider application field. Due to the characteristics of human poses and the difficulty of accurately labeling 3D ground truth, applying 3D HPE is much more difficult than applying 2D HPE^21,22^. Additionally, expensive depth cameras or LiDARs are needed during the detection process. In reference^19,20^, through multicamera shooting of the patient’s walking process, various joint points of the human body can be detected in the world coordinate system. After combining these points with the leg length of the tested person, basic gait parameters such as gait cycle time, step length and step length are detected by trigonometric functions.

### Depth estimation

In contrast to the common method of using RGB-D as the input of the neural network to achieve joint point detection, depth prediction is achieved by designing a neural network model to autonomously predict the pixel coordinates and depth feature of the joint points from an RGB image. In reference^29^, the proposed model is a multiscale deep network that first predicts a rough global output based on the entire image and then refines the output using a finer local network. In reference^30^, two parallel encoder-decoder networks are used to infer semantic and depth information, and backbone weights are shared. Finally, a geometry-aware propagation (GAP) block is proposed to improve the performance of semantic segmentation. In reference^31^, a convolutional neural network with encoding and decoding structure is designed based on the basic principle of binocular stereo vision. In reference^32^, ADDS-DepthNet is proposed. Daytime and nighttime images of the same scenes are used as inputs of the network. Although these images have different lighting conditions, their depth information is consistent. The network obtains invariant features through skip connections. To obtain private features, the daytime private feature extractors extract daytime features, and the nighttime private feature extractors extract nighttime features. Finally, private and invariant features are used to reconstruct the corresponding depth map. In reference^33^, an improved DepthNet, HR-Depth, is proposed to redesign skip connections in the network to obtain high-resolution semantic feature maps. Feature Fusion SE-Block is proposed to improve the efficiency and effectiveness of feature fusion.

## Methods

The following statement is made for this paper: (1) this study has been approved by ethics committee of School of Automation Science and Electrical Engineering of Beihang University. (2) All experiments carried out in our paper have been approved by the School of Automation Science and Electrical Engineering of Beihang University. (3) Our experiments are conducted in accordance with relevant named guidelines and regulations. (4) Informed consent was obtained from all subjects. (5) Informed consent to participate and informed consent to publish was obtained from all patients whose have identifiable images in this paper.

In this section, we will provide a detailed description, which is how to convert the RGB images obtained during human–machine system collaborative walking into geometric features. This process has four main aspects, including the neural network feature representation method, network architecture, loss function of the corresponding network structure and post processing algorithm to convert the joint points into gait parameters for the collaborative walking of the human–machine system.

### Feature representation method

There are two common feature representation methods for human pose estimation: coordinate regression and heatmap regression. In reference^14,23^, a coordinate regression method is used to take the two dimensional coordinates of each joint point of the human body as the ground truth. This ensures the input image is directly mapped through the end-to-end network to obtain the pixel coordinates of each joint point. Reference^24–26^ adopts a method based on heatmap regression and using various parts of the human body as detection targets. The probability distribution and position information of joint points are obtained by detecting the heatmap of joint points.

**Figure 1:**
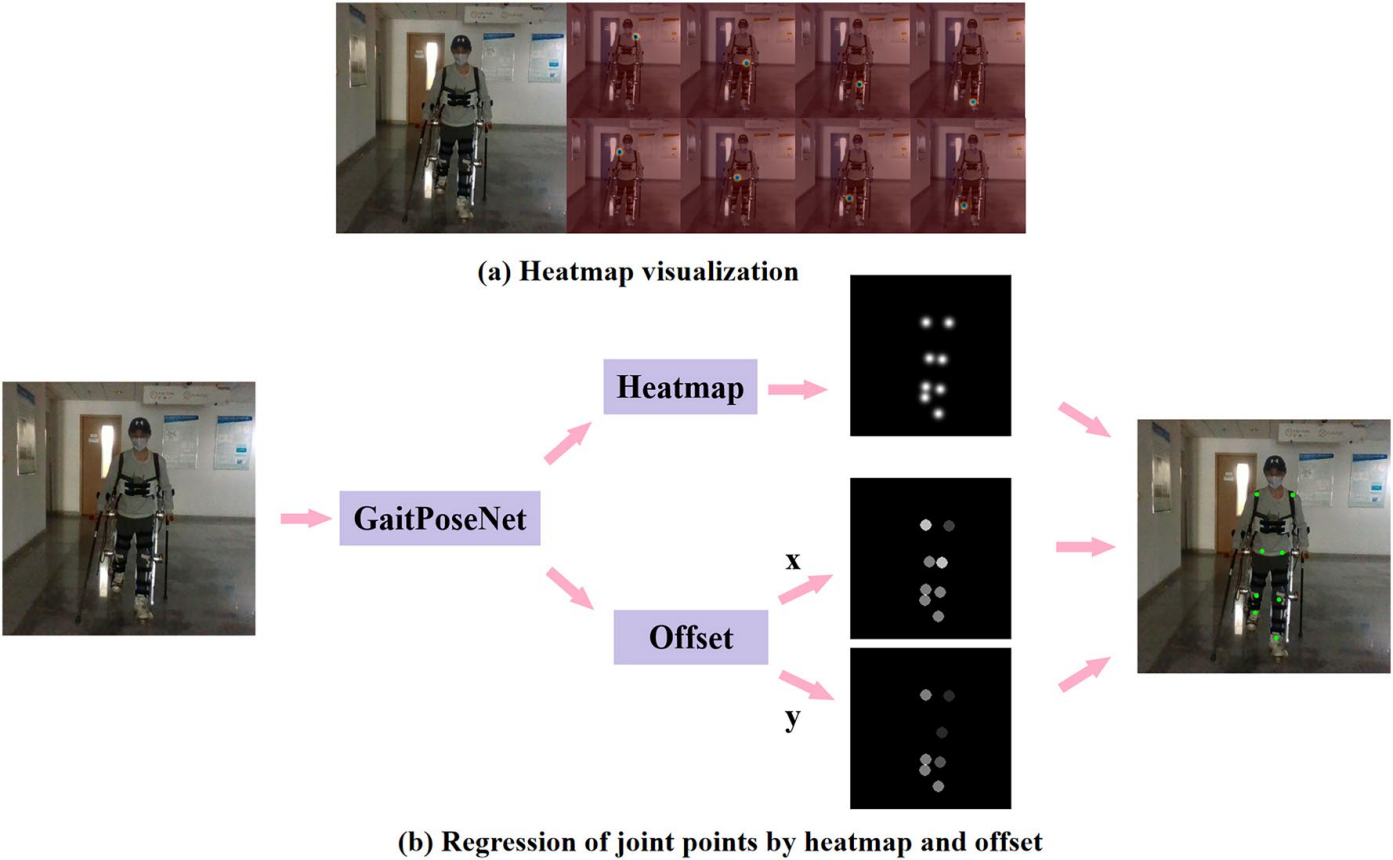
Feature representation method. (**a**) Heatmap visualization. It shows the process of generating a heatmap label by drawing a Gaussian circle with the joint point as the center. (**b**) Regression of joint points by heatmap and offset. The RGB image is used as the input of the network, and the heatmap and offset are used as the output of the network and participate in the calculation of joint point regression.

In this section, we discuss in detail the feature representation method of neural networks. As shown in Fig. 1, the neural network feature representation methods are a combination of heatmap and offset. It is difficult to accurately define many joint points by a single pixel in RGB image. If the pixels near the joint points are directly defined as negative samples, this may interfere with the training of the network, and positive and negative samples will be seriously imbalanced. Therefore, the heatmap is used to represent pixel points having joint points. The feature map size stored in the heatmap is 1/4 of the input image. A total of eight joint points are detected, and each joint point occupies one channel. Heatmap label generation is the process of generating a Gaussian circle with the joint points as the center. Traversing all the pixels of the whole image, the pixel value $H(x^p, y^p)$ of each pixel under the Gaussian distribution is as follows:

1 $$H({x^p}, y^p)=e^{-\frac{(x^p-x^p_0)+(y^p-y^p_0)}{\sigma ^2}}$$

where $(x^p_0, y^p_0)$ represents the coordinates of a certain joint point, $(x^p, y^p)$ represents any pixel on the feature map, and $\sigma$ is a constant, which we set to 6. When the value of pixel points on the feature map of the heatmap, whose pixel value is greater than 0.6, satisfy Eq. (2):

2 $$(x^p-x^p_0)^2+(y^p-y^p_0)^2\le {d_{th}}$$

where $d_{th}$ is the threshold value, which is set to 36, these pixels are identified as positive samples. The remaining pixels are regarded as negative samples.

Offset is used to characterize the pixel error generated by the process of generating heatmap labels from the input image for Gaussian heatmap restoration. The feature map size for storing offset is 1/4 of the input feature. Each joint point corresponds to two channels. Even channels predict the offset of coordinates in the x-axis direction of the joint point, while odd channels predict the offset of coordinates in the y-axis direction of the joint point. The offset label $(O^p_x, O^p_y)$ is assigned to the pixel that satisfies Eq. (2), and its value satisfies equation (3):

3 $$\begin{aligned} {\left\{ \begin{array}{ll} \ O^p_x=\frac{x^p-4x^p_h}{4}\\ \ O^p_y=\frac{y^p-4y^p_h}{4} \end{array}\right. } \end{aligned}$$

where $(x^p,y^p)$ is the pixel coordinate of a joint point, and $(x^p_h,y^p_h)$ is the coordinate of the joint point on the heatmap feature map. The resulting offset label is the normalized relative distance.

### Network architecture

**Figure 2:**
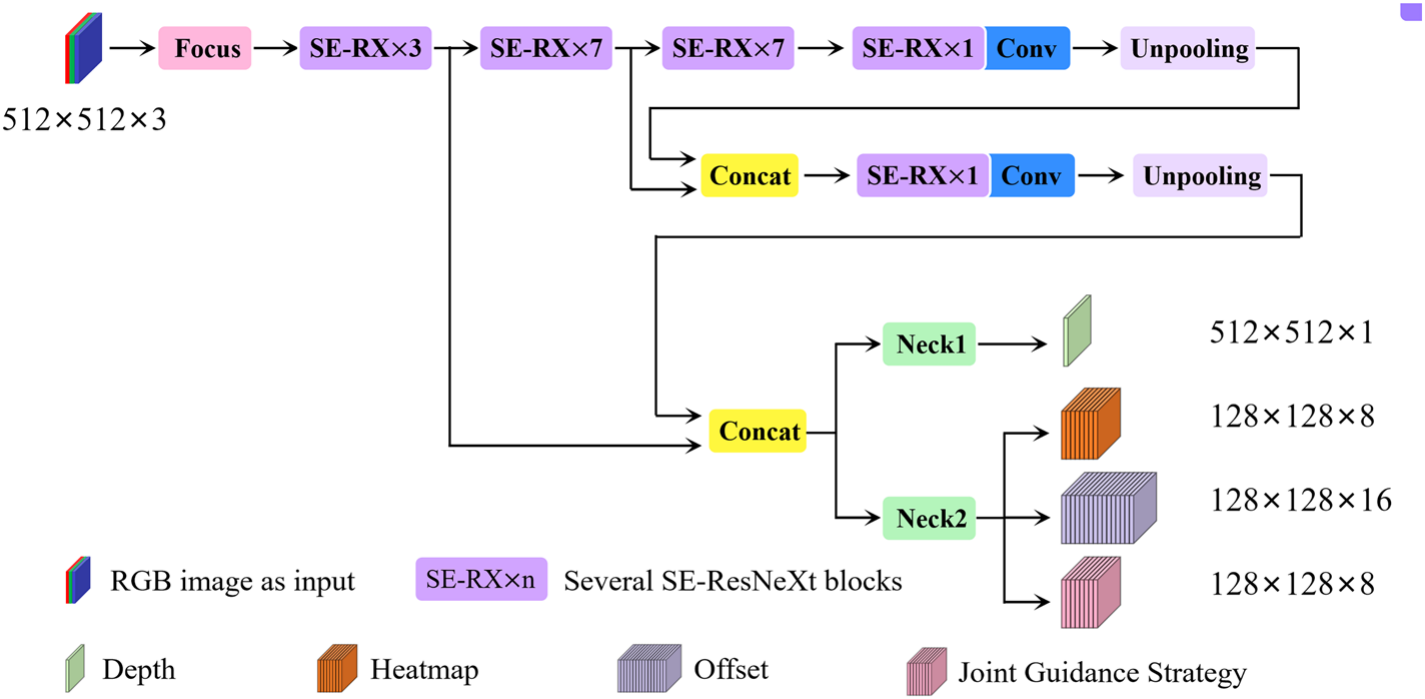
GaitPoseNet architecture. The network input is a 512 $\times$ 512 RGB image, and the output contains four branches, corresponding to tensor information that stores depth, heatmap, offset and JGS. The backbone is composed of multiple SE-ResNeXt blocks stacked together, including a focus structure and an FPN structure.

In this section, the network structure of the neural network GaitPoseNet is described in detail. As shown in Fig. 2, a 512 $\times$ 512 RGB image is used as the network input. The backbone obtains four output branches after four downsampling and two upsampling layers. The output tensor branch with size 512 $\times$ 512 $\times$ 1 is used to store depth information, the output tensor branch with size 128 $\times$ 128 $\times$ 8 is used to store heatmap, the output tensor branch with size 128 $\times$ 128 $\times$ 16 is used to store offset, and the output tensor branch with size 128 $\times$ 128 $\times$ 8 is used to store the JGS information. We use depth estimation in the network to implicitly supervise the learning of geometric information of joint points in the network. JGS is proposed to solve the problem of partial joint occlusion when a single camera captures human–machine collaborative walking. Similar to depth information, JGS only implicitly supervises the learning of the network through gradient backpropagation in the training phase and does not directly participate in the regression of joint points. The coordinates of the joint points are predicted based only on the two output branches of the heatmap and offset in inference.

Below is a detailed introduction to the network’s backbone and prediction. The backbone includes focus and feature pyramid networks (FPN), while the prediction mainly introduces the depth output end and JGS.

#### Focus module

When the image is input into the network, the input image must usually be downsampled to reduce the calculation amount and increase the receptive field. Common downsampling methods include pooling and convolution with a stride greater than 1. Both max pooling and average pooling will cause loss of details. Using convolution with a stride greater than 1 to achieve jump sampling can extract features while reducing the size of the feature map, but it will change the original features. Focus^34,35^ is essentially a tensor slicing operation that can split high resolution image into multiple low resolution image and then stack them, completely preserving image features.

#### FPN module

In CNN, the feature expression ability of feature maps differ at different levels. Shallow features mainly reflect small targets, some edge details and other information, while deep features reflect richer overall information. Therefore, FPN^36^ is introduced into the network. FPN includes bottom-up convolution, top-down upsampling and lateral connection feature fusion. It has extremely low computational cost, and it supplements deep features with shallow features through the network to compensate for the lack of geometric positioning information in high level features and obtain high resolution and strong semantic features.

#### Depth as prediction

**Figure 3:**
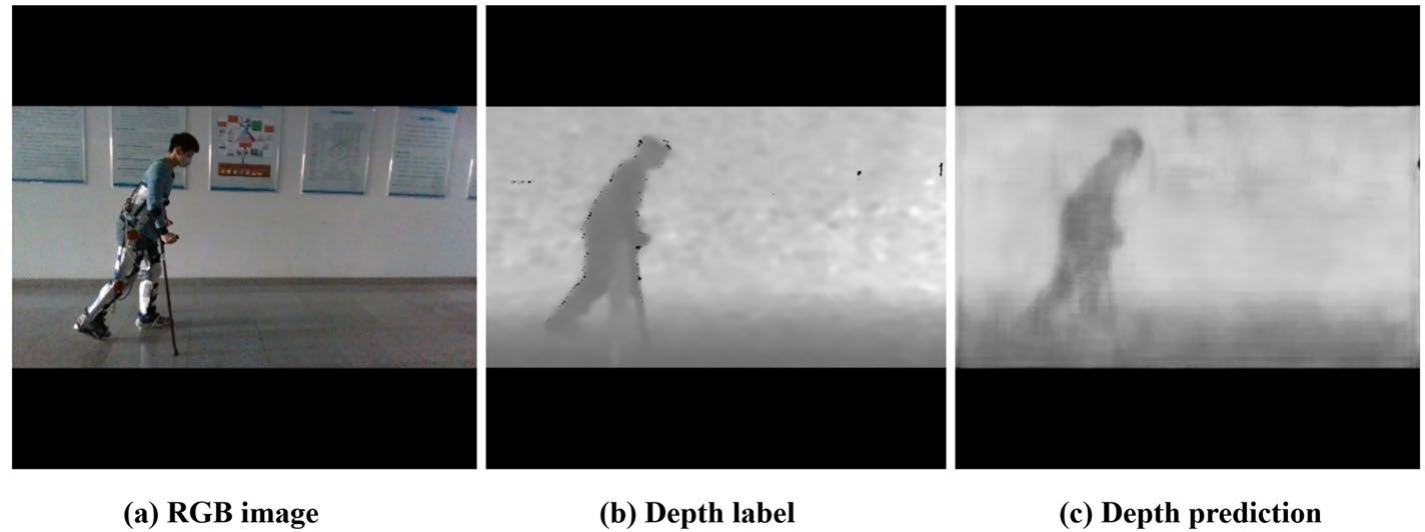
Depth as prediction. (**a**) RGB image. It shows the RGB image input of GaitPoseNet. (**b**) Depth label. It shows the depth image label corresponding to the RGB image in (**a**). (**c**) Depth prediction. It shows the depth predicted by GaitPoseNet.

The most common way to fuse multimodality is using RGB and depth modalities as inputs to the network. Usually, to better extract and fuse RGB-D features, the constructed neural network model is relatively large, and the input of the network relies on expensive devices such as depth cameras or LiDARs. The multimodality fusion model has one more depth information as input than other models, which can provide rich three dimensional structural information in the scenes. Therefore, we propose using depth information as the output of the network and using neural networks to estimate relative depth values, as shown in Fig. 3. The dimensions of the heatmap and offset feature maps are maintained in the network structure. As an important factor in understanding scene geometry, depth has the advantages of being insensitive to lighting changes, invariant to color and texture, and able to reliably estimate human body contours and skeletons. It provides the network with richer expressions for human joint points and surrounding environments. During training, depth prediction can geometrically supervise the regression of joint coordinate positions. Compared with existing 3D human pose estimation network models, our model has simpler and more convenient application scene without any depth sensors. It can predict depth independently while ensuring high quality joint detection, and the network model is simpler.

#### JGS

**Figure 4:**
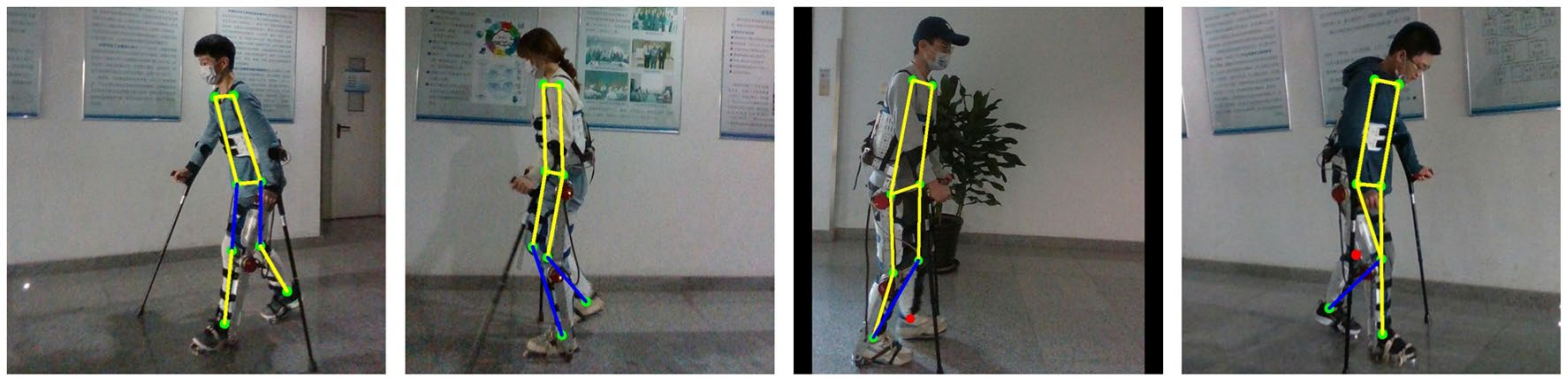
Partial joint occlusion and left-right joint matching errors during human–machine system collaborative walking. The pictures from left to right show the detection scenes of hip joint and knee joint matching errors, knee joint and ankle joint matching errors, left ankle joint missed detection, and right knee joint missed detection.

**Figure 5:**
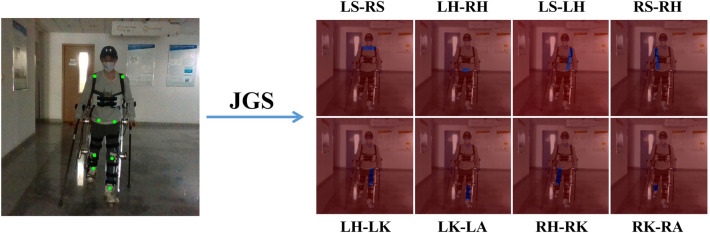
JGS labels visualization, where LS represents left shoulder, RS represents right shoulder, LH represents left hip, RH represents right hip, LK represents left knee, RK represents right knee, LA represents left ankle, and RA represents right ankle.

Figure 4 shows some problems of partial joint occlusion and left-right joint matching errors during human–machine system collaborative walking. To reduce the probability of false detection in the above situations, we added JGS to the network to predict the directional features between each pair of joints. Through unoccluded joint and the direction guidance feature, the network can be guided quickly and accurately to capture occluded joint features. The connection method of each joint in this model is shown in Fig. 5.

There are a total of 8 bone connections, and each bone connection corresponds to a channel. Therefore, the tensor size of the output feature map is 128 $\times$ 128 $\times$ 8.

**Figure 6:**
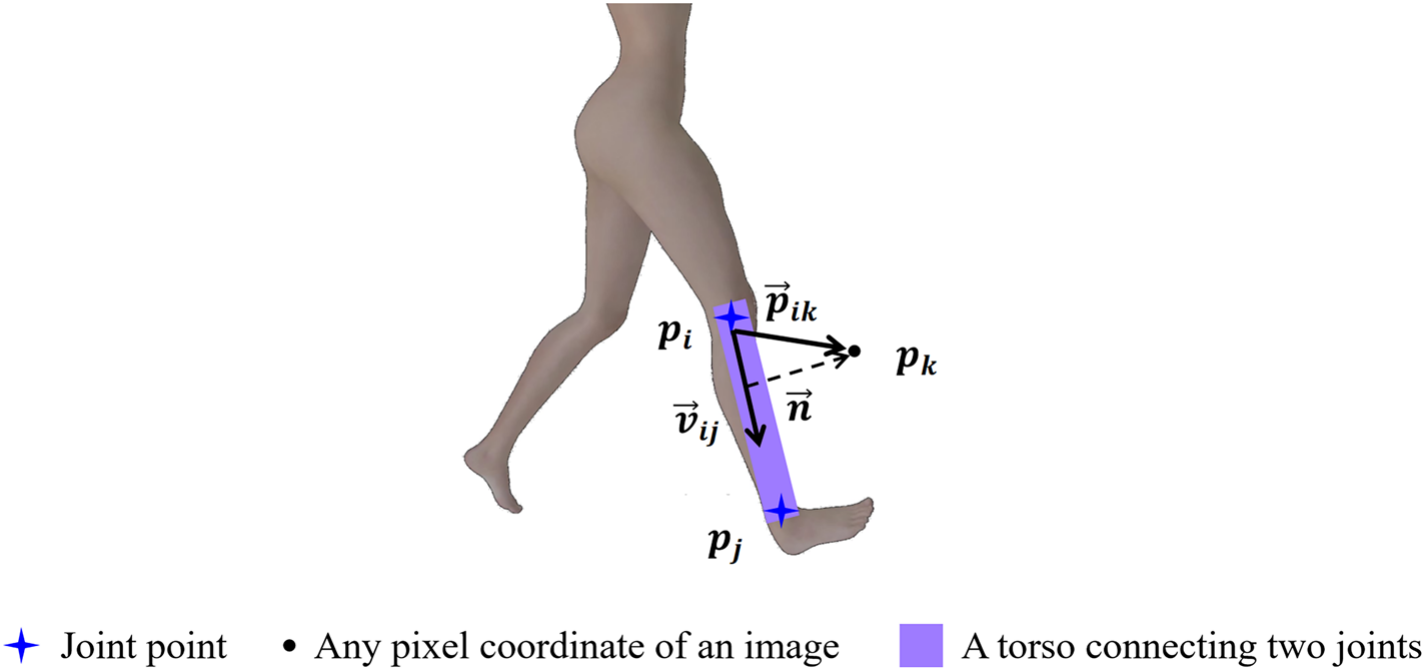
JGS for the connection between the right knee joint and the right ankle joint.

In Fig. 6, taking the connection between the right knee joint and the right ankle joint as an example, and assuming that the coordinate of the right knee joint is $p_i$ and the coordinate of the right ankle joint is $p_j$ ,then the unit vector from the right knee joint to the right ankle joint is $\vec {v}_{ij}=\frac{p_i-p_j}{||p_i-p_j||_2}$ . Therefore, for any pixel coordinate on the plane, the constraint condition for connecting the trunk between the right knee joint and the right ankle joint is as follows:

4 $$\begin{aligned} {\left\{ \begin{array}{ll} \ 0 \le {\vec {v}_{ij} \times \vec {p}_{ik}} \le {{||p_i-p_j||}_2}\\ \ \vec {n}\cdot {\vec {p}_{ik}} \le {\delta } \end{array}\right. } \end{aligned}$$

where $\vec {p}_{ik}$ is the vector from any pixel coordinate $p_k$ to the right knee joint, and $\vec {n}$ is the normal vector of $\vec {v}_{ij}$ passing through the point $p_k$. $\delta$ is the threshold set to ensure the width of the trunk, it is set to 8 here. The first constraint condition ensures that all pixel points on the trunk in the horizontal direction are found, and the second constraint condition sets the width of the trunk. By limiting these two conditions, all pixel points falling on the trunk are obtained.

### Loss function

The output of GaitPoseNet during human–machine system collaborative walking includes 4 branches. We inherited the idea of multitask loss^37^in designing the network loss function. The loss function encompasses heatmap loss, offset loss, depth loss and JGS loss. The specific formula of the function is shown below:

5 $$L(h_{ij},g_{ij},d_{ij},o_{ij})=\frac{1}{mn}\sum _{i=1}^m \sum _{j=1}^n( \alpha L_{heat}(h_{ij}, {\hat{h}}_{ij})+\beta L_{JGS}(g_{ij}, {\hat{g}}_{ij})+ L_{depth}(d_{ij}, {\hat{d}}_{ij}))+ \frac{1}{lk}\sum _{i=1}^l \sum _{j=1}^k \gamma L_{off}(o_{ij}, {\hat{o}}_{ij})$$

where m and n represent the height and width of the output tensor of heatmap, depth and JGS, respectively, both of which are 128. l and k represent the height and width of the output tensor of offset, both of which are 512. $L_{heat}$ is the loss function of the heatmap. $h_{ij}$ represents the probability of predicting the existence of a joint node at this pixel point. The mean square error (MSE) is used, and ${\hat{h}}_{ij}$ is its corresponding label. $L_{JGS}$ is the loss function of JGS. The MSE loss function is used. $g_{ij}$ represents the predicted trunk information, and ${\hat{g}}_{ij}$ is its corresponding label. $L_{depth}$ is the loss function of depth. The MSE loss function is used. $d_{ij}$ represents the output tensor that stores depth information predicted by the model, and ${\hat{d}}_{ij}$is its corresponding label. $\alpha$, $\beta$ and $\gamma$ represent the weight coefficients of heatmap loss, JGS loss and offset loss, respectively. Considering the importance of the heatmap regression task and its small numerical factors, their corresponding weight coefficients are set to 16, while the weight coefficients of JGS loss and offset loss are set to 4 and 2, respectively. For the offset loss function $L_{off}$ , $o_{ij}$ represents the offset error generated by the coordinate regression of joint nodes based on the heatmap, and ${\hat{o}}_{ij}$ is its corresponding label. MSE is also used as its loss function, and only pixels within the area satisfying Eq. (2) are used for loss function calculation.

### Gait analysis for rehabilitation assessment

In clinical examination, the process of objectively describing the functional status of patients with diseases, injuries or disabilities in their limbs and reasonably interpreting the results is called rehabilitation assessment. Gait assessment is an important part of the motor function assessment content in rehabilitation assessment^1–3^. In the rehabilitation medical scene, patients whose motor function is affected due to damage to the motor system or nervous system must undergo multiple gait assessments, which further highlight the differences and degree of differences between their gait and normal gait. This provides a basis for their further treatment plans. Based on the joint detection method during human–machine system collaborative walking introduced, this section introduces in detail the method of analyzing gait parameters by converting pixel coordinates to camera coordinates and combining patient physiological parameters. Gait parameters include joint angle parameters, gait spatiotemporal parameters and gait symmetry indicators. In the detection process, the camera must be placed such that the camera axis is perpendicular to the sagittal plane of the human body and the required detected joints are within the viewing frustum of the camera. The following provides a detailed introduction to parameter solving for joint angle parameters, gait spatiotemporal parameters and phase symmetry index.

#### Joint angle parameters

**Figure 7:**
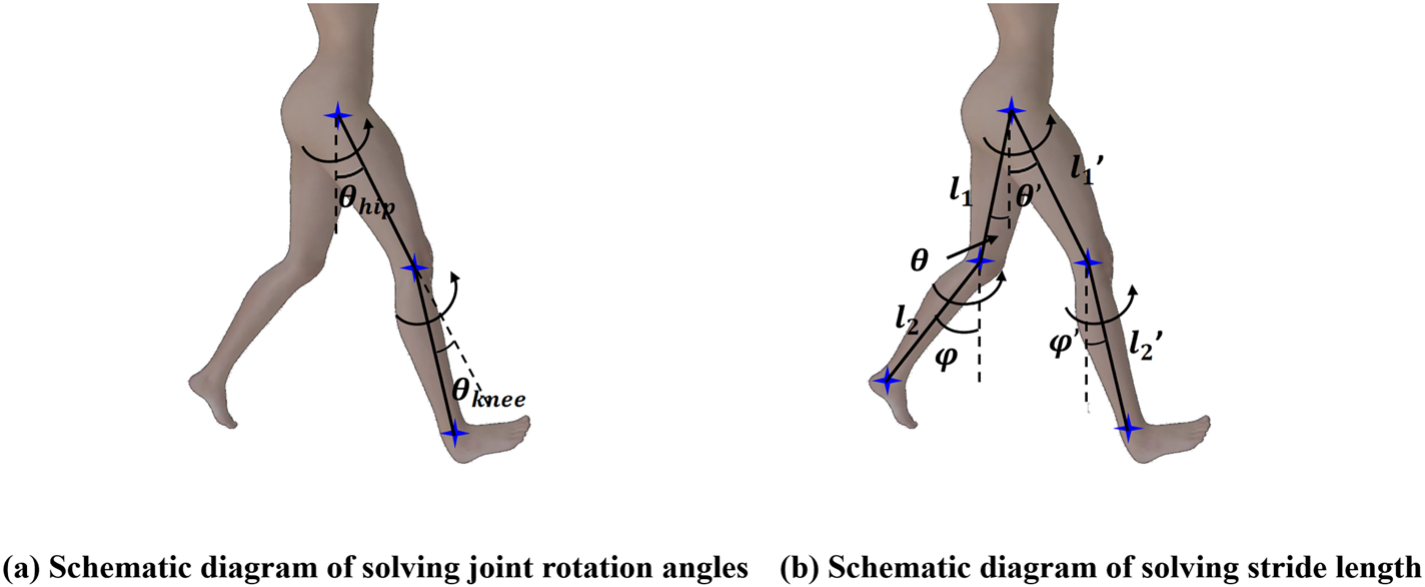
Schematic diagram of Gait analysis method. (**a**) Schematic diagram of solving joint rotation angles. (**b**) Schematic diagram of solving stride length.

In reference^38^, the hip joint rotation angle is defined as the angle between the thigh and the vertical downward direction, counterclockwise is positive, and the knee joint rotation angle is defined as the angle between the lower leg and the thigh, clockwise is positive. The pixel coordinates of a certain joint point are assumed to be $(x^p, y^p)$ , and its corresponding camera coordinate system coordinates are assumed to be $(x^c, y^c, z^c)$. The 2D coordinate to 3D coordinate transformation is implemented according to the pinhole imaging principle. The formula is as follows:

6 $$\begin{aligned} {\left\{ \begin{array}{ll} \ x^c=z^c \cdot {\frac{x^p}{f_x}} \\ \ y^c=z^c \cdot {\frac{y^p}{f_y}} \\ \ z^c=z^c \end{array}\right. } \end{aligned}$$

where $f_x$, $f_y$ are the camera focal lengths. In Fig. 7, the joint on the right side of the patient is taken as an example and the following assumptions are taken: the pixel coordinates of the right hip joint are $(x^p_1, y^p_1)$, the pixel coordinates of the right knee joint are $(x^p_2, y^p_2)$, and the pixel coordinates of the right ankle joint are $(x^p_3, y^p_3)$. Their corresponding camera system coordinates are $(x^c_1, y^c_1, z^c_1)$, $(x^c_2, y^c_2, z^c_2)$ and $(x^c_3, y^c_3, z^c_3)$ , respectively. Since the camera axis is perpendicular to the sagittal plane of the subject, $z^c_1=z^c_2=z^c_3$ .

The hip joint angle and knee joint angle are analyzed using trigonometric functions in the camera coordinate system. These formula are as follows:

7 $$\begin{aligned} \theta _{hip}= & {} arccos{\frac{|y^c_2-y^c_1|}{|| \vec {A}||_2}} \end{aligned}$$

8 $$\begin{aligned} \theta _{knee}= & {} arccos{\frac{\vec {A} \cdot \vec {B}}{{|| \vec {A}||}_2 \times {|| \vec {B}||}_2}} \end{aligned}$$

Where vectors $\vec {A}$ and $\vec {B}$ are $(x^c_2-x^c_1, y^c_2-y^c_1, 0)$ and $(x^c_3-x^c_2, y^c_3-y^c_2, 0)$ , respectively. By combining and further simplifying Eqs. (6), (7), and (8) , we obtain:

9 $$\begin{aligned} \theta _{hip}= & {} arccos{\frac{f_x(y^p_2-y^p_1)}{\sqrt{(x^p_2-x^p_1)^2+f^2_x(y^p_2-y^p_1)^2}}} \end{aligned}$$

10 $$\begin{aligned} \theta _{hip}= & {} arccos{\frac{f_y(x^p_2-x^p_1)(x^p_3-x^p_2)+f_x(y^p_2-y^p_1)(y^p_3-y^p_2)}{{\sqrt{f^2_y(x^p_2-x^p_1)^2+f^2_x(y^p_2-y^p_1)^2}} \times {\sqrt{f^2_y(x^p_3-x^p_2)^2+f^2_x(y^p_3-y^p_2)^2}}}} \end{aligned}$$

#### Gait spatiotemporal parameters

The spatiotemporal parameters of gait include stride length (m), stride cycle (s), and stride speed (m/s). The coordinates of the hip joint, knee joint, and ankle joint and the leg length of the subject are known. The solution for stride length is shown in the following formula:

11 $$D_{sl}=(l'_1 \sin \theta '+l'_2 \sin \varphi ')-(l_1 \sin \theta +l_2 \sin \varphi )$$

where $l_1$, $l'_1$ are the lengths of the left and right thighs of the subject, and $l_2$, $l'_2$ are the lengths of the left and right calves of the subject, respectively. $\theta$, $\theta '$ are the angles between the left and right thighs and the vertical downward direction of the subject, respectively, and counterclockwise is positive. $\varphi$, $\varphi '$ are the angles between the left and right calves and the vertical downward direction of the subject, respectively, and counterclockwise is positive, as shown in Fig. 7.

#### Phase symmetry index

In rehabilitation medicine, gait symmetry is an important feature characterizing human gait. In clinical practice, gait symmetry can also reveal the health status and pathological characteristics of the human body to a certain extent. High symmetry gait can reduce energy consumption and the risk of falling during human walking. In reference^39^, describing the consistency of the left and right gait functions of the human body through the phase symmetry index is proposed, and the definition formula is as follows:

12 $$D_{ps}=\sqrt{\frac{T_0}{T}}\left( T_T\frac{min(ST_L, ST_R)}{max(ST_L, ST_R)}+T_W\frac{min(SW_L, SW_R)}{max(SW_L, SW_R)}\right)$$

where $l_0$ is a constant and the statistical average value of the gait cycle of healthy people, with a unit of seconds (s). It is set to 1.2 here. *L* is the measured value of the gait cycle of the subject, with a unit of seconds (s). $ST_{L(R)}$ is the time when the left (right) legs are used as supporting legs within one walking cycle, with a unit of seconds (s). $ST_{L(R)}$ is the time when the left (right) legs are used as swing legs within one walking cycle, with a unit of seconds (s). $T_T$, $T_W$ aare the proportions of support and swing phases of each leg in the entire walking cycle when a healthy person walks normally. They are set to 0.62 and 0.38, respectively. The value range of $D_{ps}$ is [0, 1]. When the gait of the tested object is completely symmetrical, $D_{ps}$ is 1. The evaluation index range for generally healthy people is above 0.9.

## Experiments

In this section, we conduct relevant experiments to verify the accuracy and speed of the method we proposed. The progressiveness of the models are verified by comparing with the existing human posture estimation models. The accuracy of the models are verified by comparison with the HTC vive tracker, a motion capture device.

### Experimental settings

#### Dataset introduction

First, we selected 10,023 single-person images from the Microsoft Common Objects in Context (COCO) dataset^40^ and fused them with the LSP dataset^41^(containing 2000 images) to obtain a total of 12,023 RGB images of single-person poses for pretraining. To reduce the time required for data loading during training, the dataset is uniformly padded and scaled to 512 $\times$ 512. A training set of 10,001 images and a validation set of 2022 images were generated by random partitioning. In the pretraining stage, the depth map in the label is replaced by a greyscale RGB images. Most existing publicly available human pose estimation datasets are obtained from Google downloads or movie clips, and they include rich life scene information but lack multiangle shooting of images in specific scenes such as free walking of human bodies or human–machine system collaborative walking. To improve the accuracy of the model in extracting features in these scenes, we used a Realsense D435i depth camera^42^ to capture RGB images and aligned depth images of ten subjects (six males and four females) walking freely and wearing exoskeletons in different scenes. This images were captured through video. We obtained 3588 pairs of RGB-D images. The RGB-D image pairs were uniformly padded and scaled to 512 $\times$ 512 and randomly partitioned into training and test sets at a ratio of 4:1. The training set contained 2870 pairs of RGB-D images, and the validation set contained 718 pairs of RGB-D images.

The proposed model detects eight human joint points: left shoulder joint, right shoulder joint, left hip joint, right hip joint, left knee joint, right knee joint, left ankle joint and right ankle joint. Therefore, in the label setting, we use a 16 tuple to represent the coordinates of each joint in the pixel coordinate system. Each joint occupies a two-tuple that stores its x-axis coordinate and y-axis coordinate.

#### Training strategy

Deep learning models predict some values based on the patterns learnt from training data. When the number of training set samples is insufficient, the model can easily become overfit. Considering the high labor cost of producing and accurately labeling many datasets for human pose estimation, transfer learning techniques were used in the model training process. Pretraining is performed on the public COCO and LSP datasets, and then a portion of the backbone network is frozen for fine-tuning on the WPE dataset.

For the training model, we used the PyTorch framework on a workstation with an Intel i9-13900 CPU and a RTX 3090 GPU. As previously mentioned, the size of the RGB images input to the network is 512 $\times$ 512. In the pretraining stage, we trained for 100 epochs with a batch size of 8 using the Adam optimizer, with weight decay set to $10^{-6}$ , the initial learning rate set to 0.0005, and a dynamic adjustment strategy used for the learning rate. The learning rate is halved after 50 epochs of training. In the finetuning stage, we trained for 150 epochs with a batch size of 8 using the Adam optimizer and weight decay set to $10^{-6}$, but with a small learning rate set to 0.0001. The learning rate is halved every 50 epochs.

In terms of data augmentation, random occlusion, random rotation (within a range of 0$^{\circ }$–10$^{\circ }$ clockwise or counterclockwise), and random mirroring with a probability of 0.5 were used in the fine-tuning stage to increase the diversity of samples. Random occlusion is used to simulate the phenomenon of partial joint occlusion during human walking. Random rotation can compensate for camera angle tilt caused by manual shooting during dataset collection. Random mirroring with a probability of 0.5 ensures that the position of the subject in WPE dataset is evenly distributed on both sides.

#### Evaluation index

Common evaluation indicators include the percentage correct keypoints (PCKs) on head length, which are used to evaluate the accuracy of joint point detection. That is, they evaluate whether the proportion of normalized distances between the predicted value and the true value is less than the set threshold. The normalized distance often uses head width. In our model, PCK is adjusted according to the characteristics of the dataset and rewritten as:

13 $$PCKs=\frac{1}{N} \sum _{n=1}^N\left( \frac{1}{k} \sum _{k=1}^K \delta \left( \frac{||p^n_k-\hat{p}^n_k||_2}{\delta ^n}, \varepsilon \right) \right)$$

where $$\delta (x,\varepsilon )={\left\{ \begin{array}{ll} \ 1, x \le \varepsilon \\ \ 0, x >\varepsilon \\ \end{array}\right. }$$. *N* epresents the number of samples, and *K* represents the number of joint points on each sample. $p^n_k$ represents the predicted joint point pixel coordinates, and $\hat{p}^n_k$ is its corresponding label. $\sigma$ is the distance threshold, which is set to 0.1 and 0.08 in this study. $\delta ^n$ is the normalized distance, represented as:

14 $$\delta ^n=min(\max _{k}{x_k^n}-\min _{k}{x_k^n},\max _{k}{y_k^n}-\min _{k}{y_k^n})$$

#### Experimental platform

**Figure 8:**
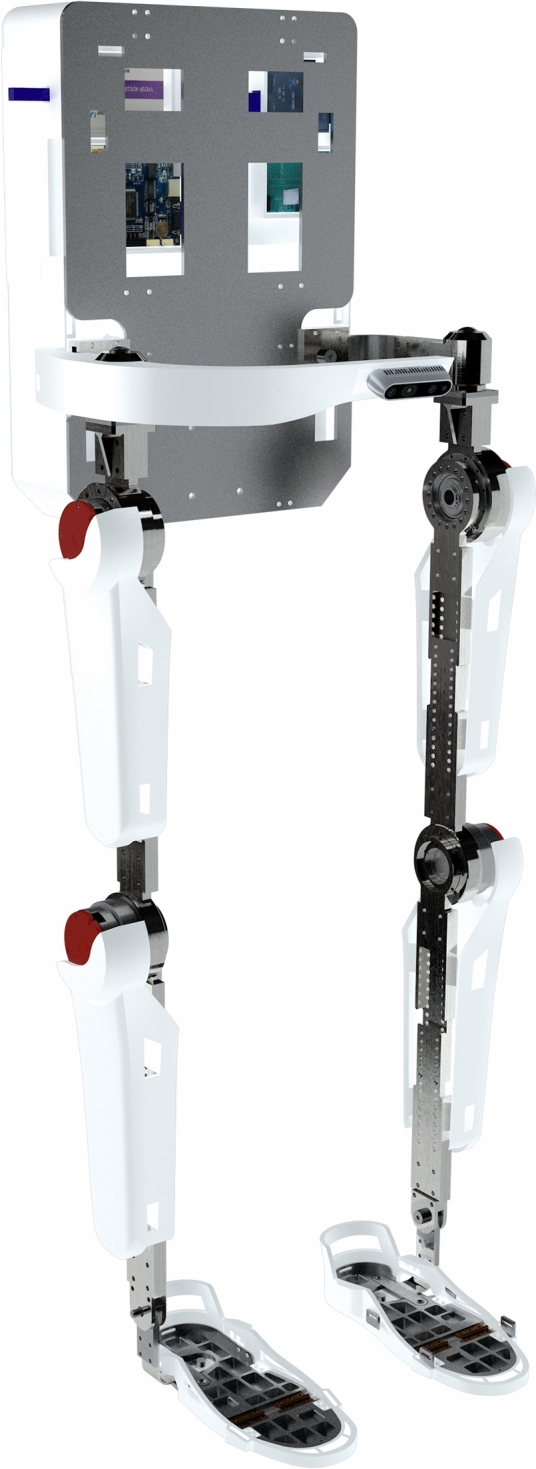
Mechanical structure of lower limb exoskeleton rehabilitation robot.

The structural diagram of the lower-limb exoskeleton rehabilitation robot we started the experiment is shown in Fig. 8. The thigh and calf dimensions of the rehabilitation lower limb exoskeleton robot are adjustable in height, which can meet the wearing requirements of a height range of 150–185 cm. The overall design is in the form of a multi link series robot. The single leg design has 5 degrees of freedom, including 1 active freedom and 2 passive fredom for the hip joint, 1 active freedom for the knee joint, and 1 passive freedom for the ankle joint.

### Experimental settings

#### Ablation experiments

To verify the influence that the focus and FPN structures proposed in “Methods” have on the network performance, we conducted the following ablation experiments. The backbone network composed of stacked SE-ResNeXt blocks is used as the baseline, and the influence of adding the focus and FPN structures on network accuracy is studied on this basis. The experimental results are shown in Table 1.

**Table 1:** Results of ablation experiments.

Backbone	Focus	FPN	PCKs@0.1 (%)	PCKs@0.08 (%)
Baseline			96.15	93.75
Baseline + Focus	$\checkmark$		96.85	94.81
Baseline + FPN		$\checkmark$	97.04	95.00
Baseline + Focus + FPN	$\checkmark$	$\checkmark$	97.18	95.27

The experimental results show that adding focus and FPN to the backbone can improve the performance of the network to a certain extent. Adding these two structures can improve the accuracy of the network, especially for higher-precision indicators.

#### Performance experiments

To meet the usage requirements of different computing devices, GaitPoseNet provides a lightweight version GaitPoseNet Lite by adjusting the width of the network channel. We conducted experiments on the validation set for GaitPoseNet Full and the lightweight version GaitPoseNet Lite, comparing the PCKs@0.1, PCKs@0.08, Params and GFlops parameters of the two models. The experimental results of the two versions of the model on desktop devices (Intel i9-13900 CPU and RTX 3090 GPU) and mobile devices (R7 7735H CPU and RTX 4060 laptop GPU) are also given in the table. The specific device models and corresponding inference speeds of each device are shown in Table 2.

**Table 2:** Results of ablation experiments.

Backbone	PCKs@0.1 (%)	PCKs@0.08 (%)	Params (M)	GFlops(M)	Runtime(Platform1) (ms)	Runtime(Platform2) (ms)
GaitPoseNet full	97.18	95.27	3.3	25.37	4.81	11.71
GaitPoseNet lite	95.77	93.14	0.8	6.54	3.55	6.79
HRNet	97.23	96.40	28.5	41.03	33.4	107.5
HRNet lite	97.37	96.57	7.4	14.83	30.3	94.6

**Figure 9:**
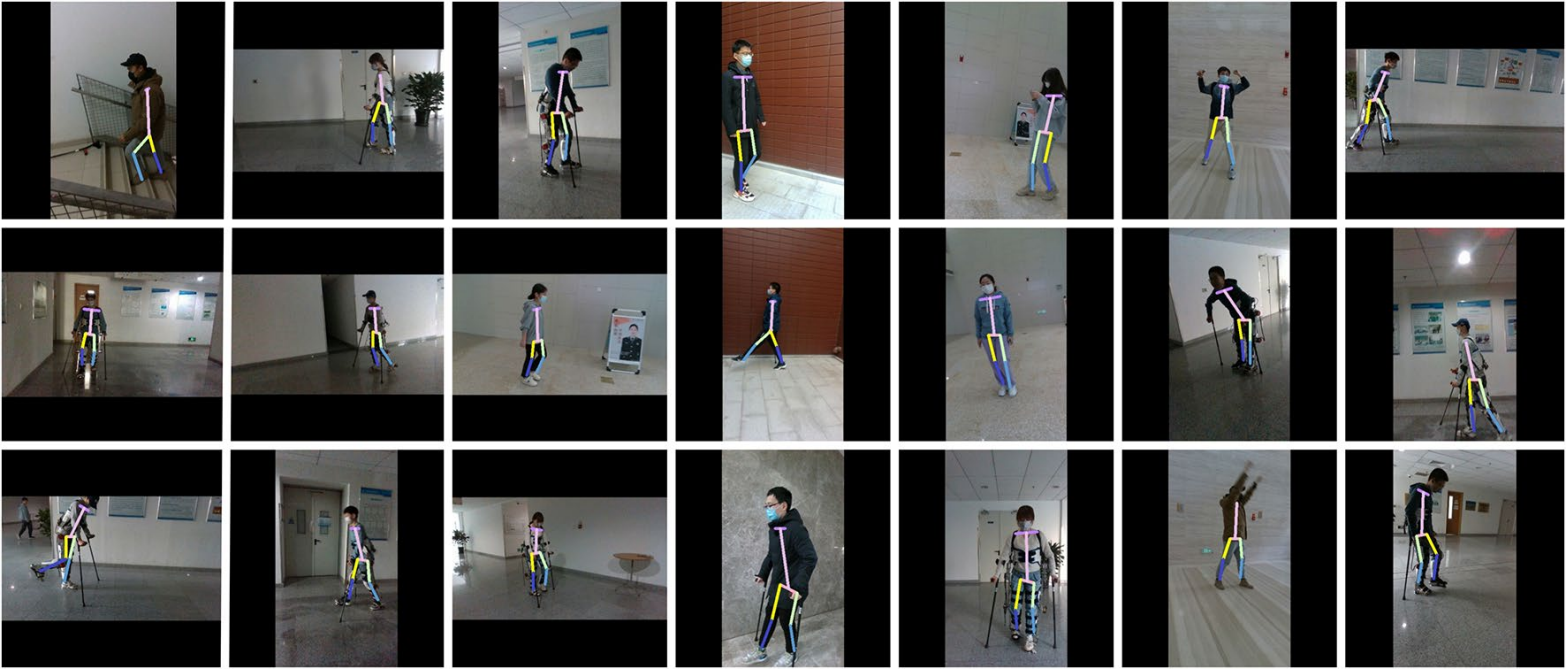
Partial validation set visualization results.

The experimental results in Table 2 show that GaitPoseNet Full and GaitPoseNet Lite have good real-time performance on desktop devices. GaitPoseNet Full performs better than GaitPoseNet Lite and is suitable for detection scene with high precision requirements. At the same time, the lightweight version model is only 1/4 the size of GaitPoseNet Full. Although its experimental accuracy is slightly lower than that of GaitPoseNet Full, it obtains a model with higher inference speed at the cost of sacrificing a small amount of accuracy. It is suitable for mobile devices and can meet application scene with high real-time requirements. The precision of the lightweight version of HRNet is higher than that of HRNet. The reason might be that HRNet is more suitable for training larger sample sizes and richer dataset, for example, COCO dataset^40^. Compared to the COCO dataset, our dataset only contains samples of various human walking scenes, and the total number of samples is lower than the COCO dataset. Compared to GaitPoseNet Full in metrics pck@0.1 and pck@0.08, HRnet Lite has increased by 0.19% and 1.30% respectively, but the number of parameters in the model is much larger than that of GaitPoseNet Full. The running time has also increases by more than nearly 9 times. Compared to GaitPoseNet Lite, the HRNet Lite performs better in terms of metrics pck@0.1 pck@0.08. It has increases by 1.60% and 3.43% , respectively, but the running time on mobile devices has also increases by more than 13 times. The visualization results on the validation set are shown in the Figure 9.

Figure 9 shows some visualized results of GaitPoseNet Full on the validation set. The results show that our model can accurately predict human posture from multiple perspectives during walking. All of our subjects are healthy. They all meet the range of normal human joint motion provided in Table 3, and there are no flexion distortion. The main basis for the inclusion of subjects is whether abnormal gait can be observed from the sagittal plane of the subjects during walking. If possible, it can be included. On the contrary, if not, our model cannot detect abnormal gait.

**Table 3:** The range of motion of various joints in the human bod.

Joint	Joint activity name	Normal walking range of motion
Hip joint	Flexion and extension	$-10^{\circ }$ to 40$^{\circ }$
	Abduction and introversion	$-3^{\circ }$ to 5$^{\circ }$
	Internal and external rotation	$-3^{\circ }$ to 7$^{\circ }$
Knee joint	Flexion and extension	0$^{\circ }$ to 67$^{\circ }$
Ankle joint	Flexion and extension	$-20^{\circ }$ to 20$^{\circ }$

#### Actual scene experiments

**Figure 10:**
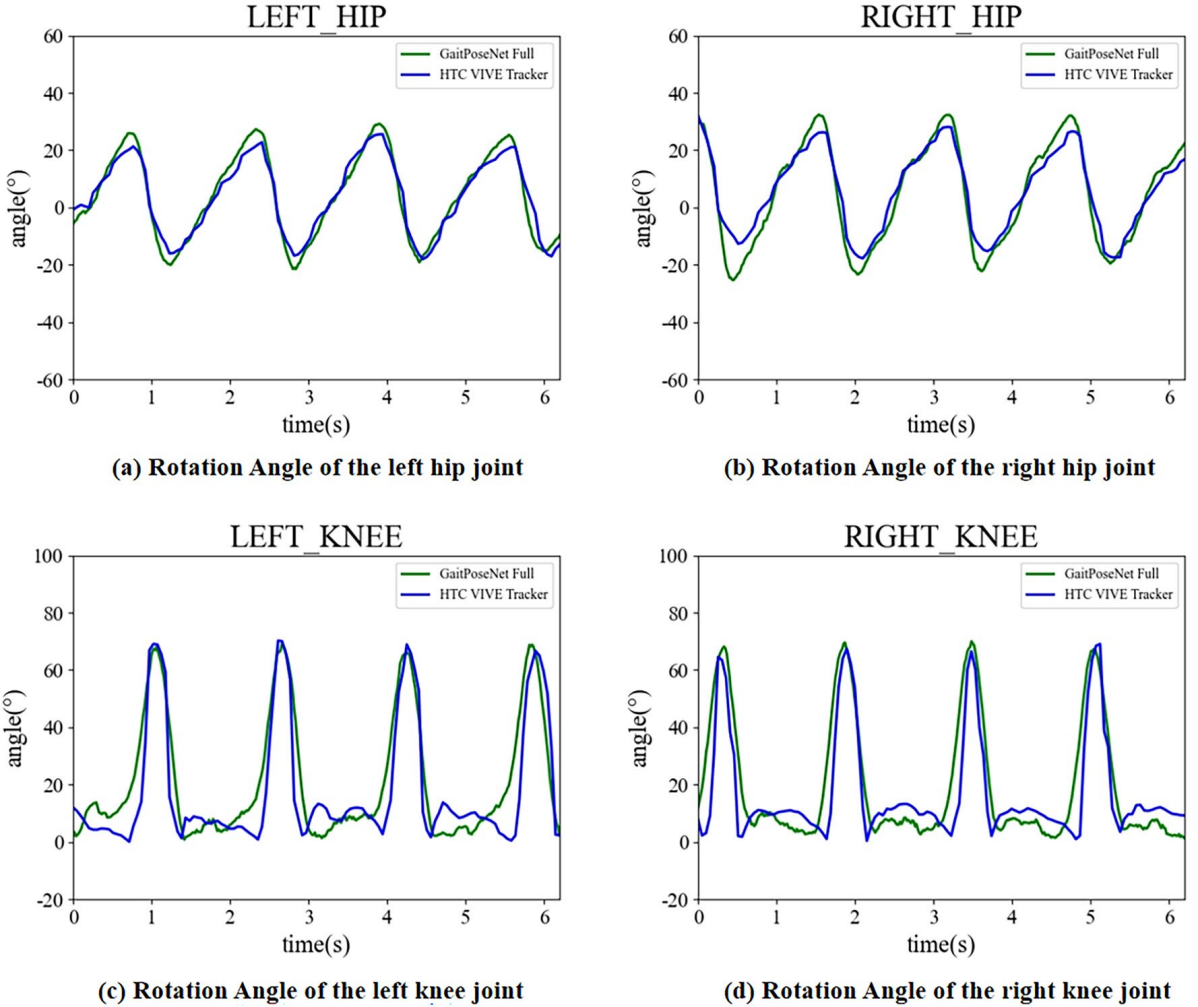
The detection results of joint rotation angles by GaitPoseNet Full and HTC vive tracker in the same scene. The image shows the left hip joint rotation angle, right hip joint rotation angle, left knee joint rotation angle, and right knee joint rotation angle from left to right and from top to bottom, respectively. The blue curve represents the HTC vive tracker detection results, and the green curve represents the GaitPoseNet Full detection results.

We use video detection to obtain higher detection accuracy. For the human–machine integrated walking scene, we obtain human walking parameters by analyzing the results of GaitPoseNet. Additionally, we analyze human motion through HTC vive tracker^43^ and analyze it from the perspectives of joint rotation angle, step length and gait symmetry. Figure 10 shows the joint rotation angles obtained through above two methods.

It can be seen that using GaitPoseNet Full and HTC vive tracker to analyze the gait results yielded joint rotation angles and trends that were very similar in Fig. 10. The video we selected is 6.2s in total, 356 frames in total. 4 gait cycles are detected, and the curve similarity is quantitatively analyzed based on the Euclidean distance method of points:

15 $$S=\frac{\sqrt{\sum _{i=0}^F{(Y_G(i)-Y_T(i))}^2}}{F}$$

where i is the current number of frames, and F is the total number of frames detected in the video. It is set to 356 here. $Y_G$ and $Y_T$ are the joint rotation angles analyzed using GaitPoseNet Full and HTC vive tracker at the current frame, respectively. The results obtained are shown in Table 4.

**Table 4:** Results of of similarity between joint rotation curve.

Quantitative analysis of similarity between joint rotation curves	Left hip	4.80
	Right hip	8.36
	Left knee	11.89
	Right knee	17.75

Table 4 shows the similarity level based on Eq. (14) using the HTC vive tracker and GaitPoseNet. Because HTC vive tracker is a contact measurement method based on laser ranging. The accuracy is extremely high. But due to the use of contact measurement, it can have a negative impact on the subject’s walking. We use the measurement results of the HTC vive tracker as the standard value. The closer the values are to 0 in Table 4, the more accurate our method measures. It is not difficult to see that the detection accuracy of the hip joint is higher than that of the knee joint. Our method is to use the camera on the left side of the human body, which will cause occlusion of the right joint. From Table 4, it can also be seen that the measurement accuracy of the unobstructed side is higher than that of the occluded side.

## Discussion

For patients with lower limb movement dysfunction wearing exoskeletons to walk, we propose a gait detection method based on computer vision technology. As a non-contact measurement method, it is used for gait evaluation of patients during rehabilitation training to provide objective and quantitative analysis. First, we designed a neural network model GaitPoseNet based on heatmap and offset regression and combined with the prediction of depth information. The depth information is used as the output and does not directly participate in the regression of the joint points. It only plays an implicit supervision role in the network regression. The advantages of being insensitive to lighting changes, invariant to color and texture, and reliably estimating human contours and skeletons are used to give the network stronger foreground and background discrimination capabilities. In addition, for the characteristics of human–machine system collaborative walking, we proposed JGS in the network structure. By predicting the connection between each joint of the patient’s trunk, the neural network is guided to capture the feature of the occluded joint according to the trunk direction based on the unoccluded joint information, thereby improving the quality of human posture estimation. It effectively solves the problem that some joint points are predicted incorrectly due to partial joint occlusion caused by shooting with a single camera. On this basis, we designed a postprocessing algorithm that regresses the pixel coordinates of the joint points combines them with the patient’s leg length to analyze gait during human–machine system collaborative walking. Gait analysis indicators include joint rotation angle, spatiotemporal parameters, gait symmetry, etc. Several experiments were conducted on the Walking-Pose with Exoskeleton dataset to evaluate the proposed method, including ablation experiments on the impact of adding Focus structure and FPN structure on network accuracy, performance experiments, and gait analysis experiments in actual scene. The experimental results show that both neural network models and postprocessing algorithms are suitable for gait analysis in this specific scene of human–machine system collaborative walking. A lightweight version of the model is also provided that can meet real-time detection requirements on mobile devices.

In the gait analysis methods for rehabilitation-type lower limb exoskeletons, it is roughly divided into detection based on wearable sensors^4–13^ and detection methods based on visual technology^14–18,44–47^. The former is a contact measurement method that negatively affects the patient’s walking movement during detection. Different sensors need to be connected to embedded devices for data transmission and reading, and professional technicians are required to analyze the data, which has poor versatility. The latter detects the position information of joint points through visual technology, reconstructs the human body trunk according to the connection between joint points, and finally analyzes gait parameters through human body structure.

With deep learning gradually becoming the mainstream method in the field of computer vision, human posture estimation technology has developed rapidly. However, 2D human posture estimation^14,15,17,18,23–28^ only uses RGB single-modal information, lacks spatial generalization ability, and is greatly affected by lighting, texture, and wrinkles of the subject’s clothing. At the same time, the most common way of existing multimodal fusion is to use RGB modality and depth modality as inputs to the network at the same time. However, the neural network model constructed is often large in size, and the input of the network needs to rely on expensive equipment such as depth cameras or lidars. Therefore, we introduce depth estimation technology to predict human joint points and depth information from single RGB modality input and use depth information to implicitly supervise joint point regression. In future work, we want to further improve the neural network structure and directly regress the coordinates of joint points in the world coordinate system by learning depth information.

## Conclusion

We propose a neural network GaitPoseNet dedicated to joint point detection, which uses the combined method of heatmap and offset to regress joint point coordinates. By adding depth prediction and JGS implicit supervision network to the output end of the network, the addition of depth prediction can improve the network’s ability to distinguish foreground and background, and the addition of JGS can guide the network to find features of occluded joint points through trunk information. Our designed post-processing algorithm can realize a complete gait detection system for human–machine system collaborative walking based on the pixel coordinates of 8 detected joint points combined with the subject’s leg length. Through this method, it is possible to achieve non-contact monitoring of gait during patient rehabilitation training. Lightweight version GaitPoseNet Lite’s specific data on PCKs@0.1 and PCKs@0.08 can reach 95.77% and 93.14%. And the runtime can reach 3.55 ms per frame, which fully meets the requirements for applications on mobile laptops. The experimental results demonstrate that our method achieves faster detection at the expense of small accuracy. Due to the fact that we output relative depth information, we are unable to accurately predict the true depth image. Therefore, our method can only detect the information of the subject in the forward direction, and cannot detect the behavior of the subject in the left and right directions. In addition, for the study of human gait, we provide a human walking posture dataset Walking-Pose with Exoskeleton with depth maps and fine annotations. In future work, we will further improve the depth estimation algorithm to enable the neural network to regress depth information more accurately and realize gait analysis in the world coordinate system for human–machine system collaborative walking and turning.

## Data Availability

Our dataset is available at https://doi.org/10.17632/637s7mvsg7.1. And datasets for pretraining are available at COCO dataset (https://doi.org/10.1007/978-3-319-10602-1_48) and LSP dataset (https://doi.org/10.48550/arXiv.1605.01014).

## References

[1] Zhu, A., Tu, Y., Zheng, W., Shen, H. & Zhang, X. Adaptive control of man-machine interaction force for lower limb exoskeleton rehabilitation robot. In *2018 IEEE International Conference on Information and Automation (ICIA)*, 740–743. 10.1109/ICInfA.2018.8812503 (2018).

[2] Gan, D., Qiu, S., Guan, Z., Shi, C. & Li, Z. Development of a exoskeleton robot for lower limb rehabilitation. In *2016 International Conference on Advanced Robotics and Mechatronics (ICARM)*, 312–317. 10.1109/ICARM.2016.7606938 (2016).

[3] Yuan, Y., Cao, G.-Z., Zhu, A., Lyu, X. & Wang, Y. Communication scheme of cloud platform for the lower limb exoskeleton rehabilitation robot. In *2020 17th International Conference on Ubiquitous Robots (UR)*, 327–332. 10.1109/UR49135.2020.9144989 (2020).

[4] Monoli C (2021). Land and underwater gait analysis using wearable imu. IEEE Sens. J..

[5] Wang L, Sun Y, Li Q, Liu T, Yi J (2020). Two shank-mounted imus-based gait analysis and classification for neurological disease patients. IEEE Robot. Autom. Lett..

[6] Wang L, Sun Y, Li Q, Liu T, Yi J (2021). Imu-based gait normalcy index calculation for clinical evaluation of impaired gait. IEEE J. Biomed. Health Inform..

[7] Lee Junhee BCH, Jang Aeri YS, Hasuk B (2020). Determining the most appropriate assistive walking device using the inertial measurement unit-based gait analysis system in disabled patients. Ann. Rehabil. Med..

[8] Qin, L.-y., Ma, H. & Liao, W.-H. Insole plantar pressure systems in the gait analysis of post-stroke rehabilitation. In *2015 IEEE International Conference on Information and Automation*, 1784–1789. 10.1109/ICInfA.2015.7279576 (2015).

[9] Li, B. *et al.* Foot plantar pressure measurement system based on flexible force-sensitive sensor and its clinical application. In *2018 IEEE 3rd Advanced Information Technology, Electronic and Automation Control Conference (IAEAC)*, 1998–2002. 10.1109/IAEAC.2018.8577945, (2018).

[10] Shi, C. *et al.* Design of plantar pressure monitor system of exoskeleton assistant device. In *2014 IEEE International Conference on Mechatronics and Automation*, 1649–1653. 10.1109/ICMA.2014.6885947 (2014).

[11] Wang, J., Dai, Y., Kang, T. & Si, X. Research on gait recognition based on lower limb emg signal. In *2021 IEEE International Conference on Mechatronics and Automation (ICMA)*, 212–217. 10.1109/ICMA52036.2021.9512759 (2021).

[12] Kim, Y.-h., Kim, S.-j., Shim, H.-m., Lee, S.-m. & Kim, K.-s. A method for gait rehabilitation training using emg fatigue analysis. In *2013 International Conference on ICT Convergence (ICTC)*, 52–55. 10.1109/ICTC.2013.6675305 (2013).

[13] Ryu J, Kim DH (2017). Real-time gait subphase detection using an emg signal graph matching (esgm) algorithm based on emg signals. Expert Syst. Appl..

[14] Pishchulin, L. *et al.* Deepcut: Joint subset partition and labeling for multi person pose estimation. In *2016 IEEE Conference on Computer Vision and Pattern Recognition (CVPR)*, 4929–4937. 10.1109/CVPR.2016.533 (2016).

[15] Hua G, Li L, Liu S (2020). Multipath affinage stacked-hourglass networks for human pose estimation. Front. Comput. Sci..

[16] Wang J (2021). Deep high-resolution representation learning for visual recognition. IEEE Trans. Pattern Anal. Mach. Intell..

[17] Cao, Z., Hidalgo, G., Simon, T., Wei, S.-E. & Sheikh, Y. Openpose: Realtime multi-person 2d pose estimation using part affinity fields (2019). arXiv:1812.08008v2. 10.1109/TPAMI.2019.2929257 31331883

[18] Fang, H.-S., Xie, S., Tai, Y.-W. & Lu, C. Rmpe: Regional multi-person pose estimation. In *2017 IEEE International Conference on Computer Vision (ICCV)*, 2353–2362. 10.1109/ICCV.2017.256 (2017).

[19] Zago M (2020). 3d tracking of human motion using visual skeletonization and stereoscopic vision. Front. Bioeng. Biotechnol..

[20] Pasinetti S (2020). Validation of marker-less system for the assessment of upper joints reaction forces in exoskeleton users. Sensors.

[21] G, U. K., V, S., Ch, N., B, G. C. & K, Y. K. Estimating 3d human pose using point based pose estimation and single stage method. In *2022 3rd International Conference on Computing, Analytics and Networks (ICAN)*, 1–5. 10.1109/ICAN56228.2022.10007130 (2022).

[22] Fang, Z., Wang, A., Bu, C. & Liu, C. 3d human pose estimation using rgbd camera. In *2021 IEEE International Conference on Computer Science, Electronic Information Engineering and Intelligent Control Technology (CEI)*, 582–587. 10.1109/CEI52496.2021.9574486 (2021).

[23] Bazarevsky, V. *et al.* Blazepose: On-device real-time body pose tracking (2020). arXiv:2006.10204.

[24] Tompson, J., Jain, A., LeCun, Y. & Bregler, C. Joint training of a convolutional network and a graphical model for human pose estimation (2014). arXiv:1406.2984v1.

[25] Wang, Z. *et al.* A light-weighted network for facial landmark detection via combined heatmap and coordinate regression. In *2019 IEEE International Conference on Multimedia and Expo (ICME)*, 314–319. 10.1109/ICME.2019.00062 (2019).

[26] Keskin, C. *et al.* Repose: Learning deep kinematic priors for fast human pose estimation. 10.48550/arXiv.2002.03933 (2020).

[27] Iqbal U, Gall J, Hua G, Jégou H (2016). Multi-person pose estimation with local joint-to-person associations. Computer Vision—ECCV 2016 Workshops.

[28] Ye M, Yang C, Stankovic V, Stankovic L, Cheng S (2020). Distinct feature extraction for video-based gait phase classification. IEEE Trans. Multimed..

[29] Eigen, D. & Fergus, R. Predicting depth, surface normals and semantic labels with a common multi-scale convolutional architecture. In *2015 IEEE International Conference on Computer Vision (ICCV)*, 2650–2658. 10.1109/ICCV.2015.304 (2015).

[30] Jiao, J. *et al.* Geometry-aware distillation for indoor semantic segmentation. In *2019 IEEE/CVF Conference on Computer Vision and Pattern Recognition (CVPR)*, 2864–2873. 10.1109/CVPR.2019.00298 (2019).

[31] Eigen, D., Puhrsch, C. & Fergus, R. Depth map prediction from a single image using a multi-scale deep network. 10.48550/arXiv.1406.2283 (2014). arXiv:1406.2283.

[32] Liu, L., Song, X., Wang, M., Liu, Y. & Zhang, L. Self-supervised monocular depth estimation for all day images using domain separation. In *2021 IEEE/CVF International Conference on Computer Vision (ICCV)*, 12717–12726. 10.1109/ICCV48922.2021.01250 (2021).

[33] Lyu, X. *et al.* Hr-depth: High resolution self-supervised monocular depth estimation. 10.48550/arXiv.2012.07356 (2020). arXiv:2012.07356.

[34] Redmon, J. & Farhadi, A. Yolo9000: Better, faster, stronger. 10.48550/arXiv.1612.08242 (2016). arXiv:1612.08242.

[35] Ultralytics. Yolov5 (2019). https://github.com/ultralytics/yolov5.

[36] Zhang, Y., Han, J. H., Kwon, Y. W. & Moon, Y. S. A new architecture of feature pyramid network for object detection. In *2020 IEEE 6th International Conference on Computer and Communications (ICCC)*, 1224–1228. 10.1109/ICCC51575.2020.9345302 (2020).

[37] Nada, K., Imoto, K., Iwamae, R. & Tsuchiya, T. Multitask learning of acoustic scenes and events using dynamic weight adaptation based on multi-focal loss. In *2021 Asia-Pacific Signal and Information Processing Association Annual Summit and Conference (APSIPA ASC)*, 1156–1160 (2021).

[38] Xueyan Hu XY, Zhongwu Guo GW, Ding H (2006). Basic gait characteristics of healthy adults. Chin. J. Rehabil. Theory Pract..

[39] Wang R, Zhang M (2011). Comparative study on relative proportionality evaluation indicators of human gait. Chin. J. Rehabil. Med..

[40] Lin T-Y, Fleet D, Pajdla T, Schiele B, Tuytelaars T (2014). Microsoft coco: Common objects in context. Computer Vision—ECCV 2014.

[41] Yu, X., Zhou, F. & Chandraker, M. Deep deformation network for object landmark localization. 10.48550/arXiv.1605.01014 (2016). arXiv:1605.01014.

[42] Intel. Depth camera d435i. https://www.intelrealsense.com/depth-camera-d435i/.

[43] HTC. Vive tracker. https://www.vive.com/cn/accessory/tracker3/.

[44] Wang Y, Mori G, Forsyth D, Torr P, Zisserman A (2008). Multiple tree models for occlusion and spatial constraints in human pose estimation. Computer Vision—ECCV 2008.

[45] Dantone M, Gall J, Leistner C, Van Gool L (2014). Body parts dependent joint regressors for human pose estimation in still images. IEEE Trans. Pattern Anal. Mach. Intell..

[46] Yang Y, Ramanan D (2011). Articulated pose estimation with flexible mixtures-of-parts. CVPR.

[47] Krizhevsky A, Sutskever I, Hinton G (2017). Imagenet classification with deep convolutional neural networks. Commun. ACM.

